# Reply: factor II/X-guided warfarin monitoring for congenital factor VII deficiency after mechanical valve replacement

**DOI:** 10.1186/s12959-026-00923-z

**Published:** 2026-09-28

**Authors:** Xiaokang Luo, Xinjian Yan

**Affiliations:** 1 Department of Cardiovascular Surgery, Guangdong Provincial People’s Hospital, Guangdong Academy of Medical Sciences, Southern Medical University, Guangzhou, China; 2 Guangdong Cardiovascular Institute, Guangdong Provincial People’s Hospital, Guangdong Academy of Medical Sciences, No. 106 Zhongshan 2nd Road, Yuexiu District, Guangzhou, 510080 China

Dear Editors,

We sincerely appreciate the thoughtful commentary submitted by the readers regarding our recently published case report describing anticoagulation management after mechanical heart valve (MHV) implantation in a patient with congenital factor VII deficiency (CFⅦD). The commentary highlights critical challenges in anticoagulation monitoring, and we welcome this opportunity to further elaborate on the rationale underpinning our factor II and X (FⅡ/FⅩ)-guided anticoagulation strategy.

Even in patients with congenital factor VII deficiency, direct oral anticoagulants (DOACs) are not a viable alternative to warfarin for those undergoing MHV replacement. Unlike warfarin, which inhibits multiple vitamin K-dependent coagulation factors, DOACs exert selective single-target inhibition [1]: dabigatran directly suppresses thrombin (FⅡa), whereas rivaroxaban and apixaban inhibit activated factor X (FⅩa). Despite their favorable pharmacokinetic profiles across many thromboembolic indications, pivotal clinical trial data contraindicate their use in MHV recipients. The RE-ALIGN trial was terminated prematurely owing to significantly higher risks of thromboembolism and major bleeding in patients treated with dabigatran compared with warfarin [2]. Mechanistically, mechanical prostheses trigger robust contact pathway activation and sustained thrombin generation, which cannot be adequately controlled by single-target anticoagulant monotherapy. Consequently, warfarin remains the only guideline-endorsed anticoagulant for lifelong thromboprophylaxis after mechanical valve replacement [3].

The conventional INR equally weighs the circulating activities of FⅡ, FⅦ and FⅩ, yet warfarin’s antithrombotic efficacy primarily relies on suppression of the longer-lived FⅡ and FⅩ [4]. Most clinically irrelevant fluctuations in PT/INR arise from the disproportionate impact of short-half-life FⅦ [5]. In patients with CFⅦD, baseline FⅦ depletion dominates prothrombin time results, producing falsely elevated INR values that do not reflect true anticoagulation intensity. In our patient, postoperative INR rose to 4.1–4.3 relative to a preoperative baseline of 3.78. This spuriously elevated INR falsely indicated persistent bleeding risk, leading to delayed anticoagulation initiation until postoperative day 6 and resulting in acute cerebral thromboembolism. This adverse event demonstrates that INR values are dissociated from true anticoagulation intensity in CFⅦD and can contribute to life-threatening clinical misjudgements. In our case, therapeutically dosed warfarin suppressed FⅡ/FⅩ activity to our target range of 30–45%, with follow-up INR values ranging from 6.0 to 8.0. These elevated INR values were observed in the absence of any bleeding manifestations, further confirming the limited clinical utility of INR in this unique patient population.

For all mechanical heart valve recipients, INR-guided warfarin therapy carries substantial inherent practical drawbacks. The narrow therapeutic window and frequent INR variability necessitate repeated testing and dose adjustments, imposing considerable financial and time burdens on patients [6]. Suboptimal time in therapeutic range (TTR) is well established as an independent risk factor for both thromboembolic complications and major haemorrhage. These challenges heighten the clinical importance of optimizing warfarin monitoring approaches. Monitoring strategies based on FⅡ and FⅩ activity (Fiix-PT/Fiix-NR) provide a biologically plausible and clinically promising alternative [7]. This assay eliminates confounding signals originating from variable FⅦ levels and directly reflects warfarin’s core antithrombotic effect. Existing real-world data demonstrate that Fiix-NR monitoring reduces thromboembolic events by approximately 56% without elevating bleeding risk, while reducing the frequency of laboratory testing and dose adjustments [5]. While large prospective trials focused on MHV populations have yet to be conducted, our case supports the feasibility of FⅡ/FⅩ-targeted anticoagulation for balancing haemorrhagic and thrombotic risk, especially for patients in whom INR is unreliable.

We hope our report encourages further investigation into FⅡ/FⅩ-based monitoring in mechanical valve patient populations, both with and without inherited coagulation disorders.

## Data Availability

No datasets were generated or analysed during the current study.
